# Supplementary figures and images for: A proportion of CD4+ T cells from patients with chronic Chagas disease undergo a dysfunctional process, which is partially reversed by benznidazole treatment
Source: PLoS Negl Trop Dis. 2021 Feb 4;15(2):e0009059. doi: 10.1371/journal.pntd.0009059 (PMC7888659; doi:10.1371/journal.pntd.0009059)

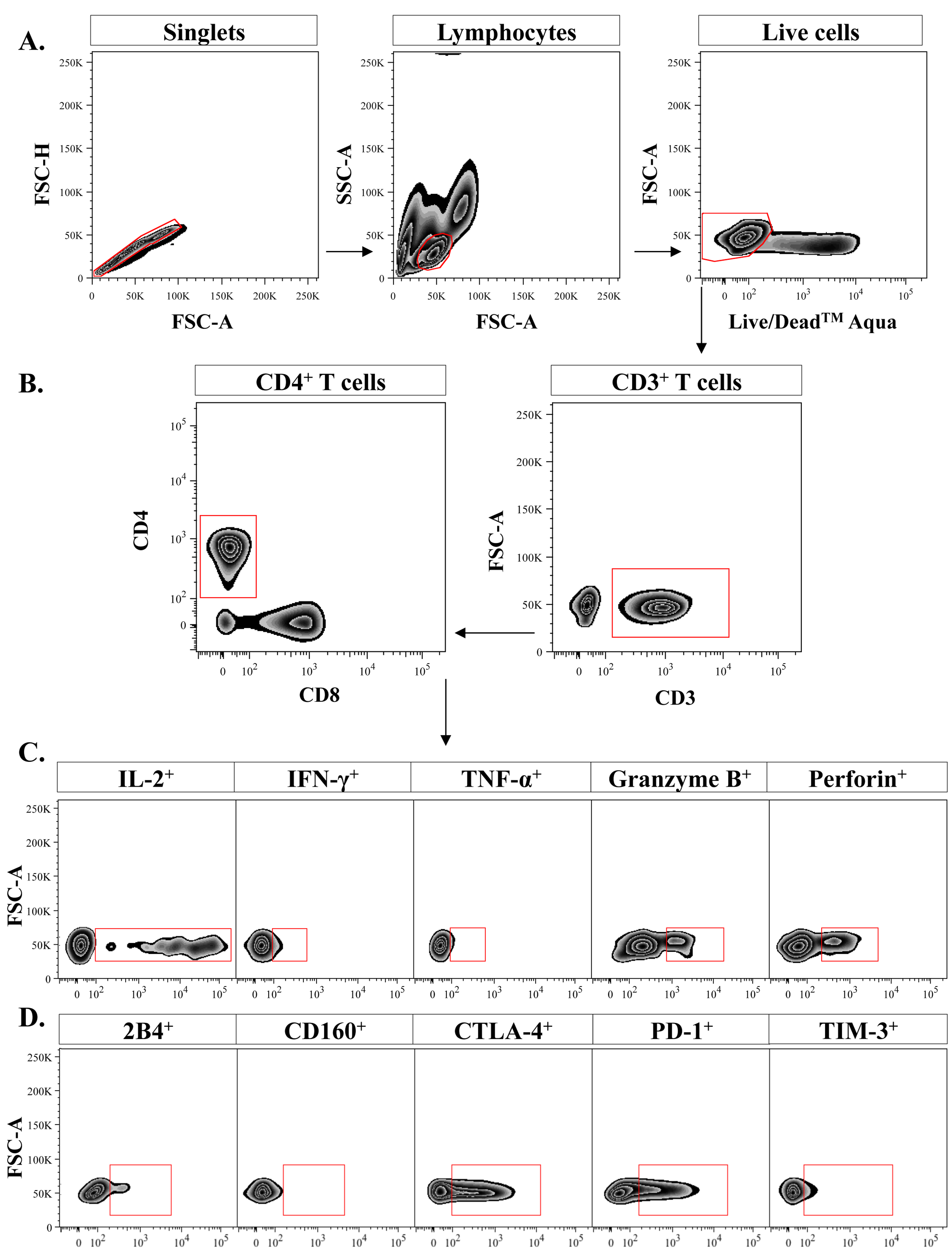

Supplement: S1 Fig — From left to right: A) Selection of singlets was performed on a dot plot that faces height versus area (FSC-H/FSC-A). Single lymphocytes were gated using FSC and SSC parameters. Subsequently, live cells were selected by its negative fluorescence after LIVE/DEAD Aqua dead cell staining. (B) Selection of CD3+ cells among the CD4+CD8-. (C) Gating strategy for selection of antigen-specific CD4+ T cells expressing IL-2, IFN-γ and TNF-α cytokines or granzyme B and perforine cytotoxic molecules. (D) Gates to assess the frequency of CD4+ T cells expressing inhibitory receptors (2B4, CD160, CTLA-4, PD-1 and TIM-3). The analyses were performed using the FlowJo 9.3.2 software. The selection of positivity for each marker was selected based on the fluorescence minus one (FMO) control, and the unstained control. (TIF) [file pntd.0009059.s001.tif]

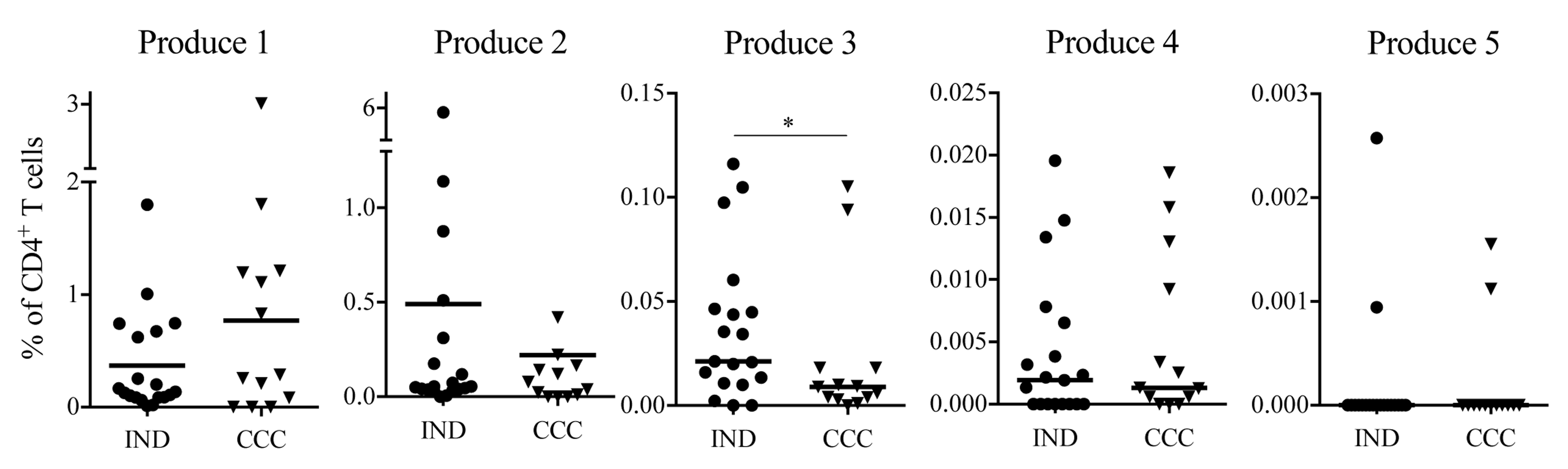

Supplement: S2 Fig — The frequency of T. cruzi antigen-specific CD4+ T cells that express or coexpress different numbers of molecules of IFN-γ, IL-2, TNF-α, perforin and/or granzyme B was evaluated in 19 indeterminate patients (IND) and 13 patients with cardiac manifestations (CCC). The coexpression analyses were performed using a Boolean gates strategy, of the five molecules under study. The data shown were obtained after stimulation with T. cruzi soluble antigens (TcSA) plus CD28 and CD49d for 10 h, subtracting the background value (cells cultured with CD28 and CD49d co-stimulators). The lines included in the graphs represent the mean for each group of data. Statistical analyzes were performed using the Mann-Whitney U test. The p values are indicated by asterisks (p<0.05 (*)). (TIF) [file pntd.0009059.s002.tif]

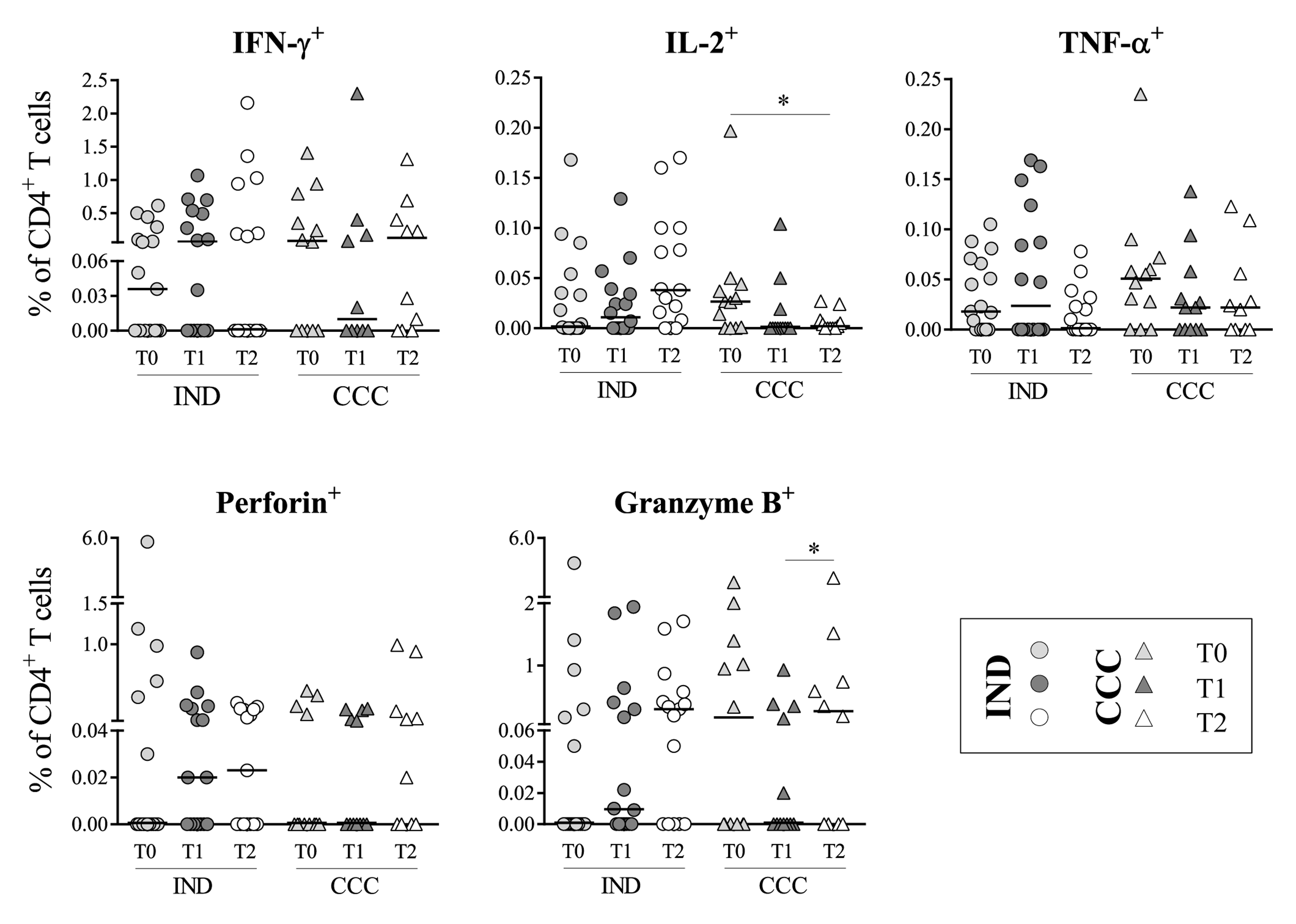

Supplement: S3 Fig — Frequency of TcSA-estimulated CD4+ T cells producing the IFN-γ, IL-2, and TNF-α cytokines, or the perforin and granzyme B cytotoxic molecules, from19 indeterminate patients (IND) and 13 Chagas disease patients with cardiac alterations (CCC) before treatment (T0); from 17 out of 19 (IND) and 12 out of 13 (CCC) at 9–12 (T1) and 24–48 (T2) months after treatment. The data shown were obtained after stimulation of cells with T. cruzi soluble antigens (TcSA) plus CD28 and CD49d for 10 h and subtracting to this value the background value (cells cultured solely with CD28 and CD49d). Statistical analyzes were performed using the Wilcoxon test. Statistically significant differences (p<0.05) are indicated with an asterisk (*). Scatter plots showing the median values represented with horizontal lines. (TIF) [file pntd.0009059.s003.tif]
