# Supplementary material for: A proportion of CD4+ T cells from patients with chronic Chagas disease undergo a dysfunctional process, which is partially reversed by benznidazole treatment
Source: PLoS Negl Trop Dis. 2021 Feb 4;15(2):e0009059. doi: 10.1371/journal.pntd.0009059 (PMC7888659; doi:10.1371/journal.pntd.0009059)
Supplement: S1 Table — (PDF) [file pntd.0009059.s004.pdf]

Variables	cChD			
	T1		T2	
	Spearman’s (ρ)	p-value	Spearman’s (ρ)	p-value
**2B4**				
T0	0.32	0.08	0.37	0.04 *
T1	-	-	0.63	0.0002 ***
**CD160**				
T0	0.50	0.005 **	0.59	0.001 **
T1	-	-	0.64	0.0002 ***
**CTLA-4**				
T0	0.38	0.04 *	0.56	0.001 **
T1	-	-	0.53	0.002 **
**PD-1**				
T0	0.35	0.06	0.32	0.09
T1	-	-	0.44	0.01 **
**TIM-3**				
T0	0.62	0.0003 ***	0.63	0.0002 ***
T1	-	-	0.77	0.000001 ****

cChD → Chronic Chagas disease patients

(ρ) → Spearman's rank correlation coefficient

p values → $p<0.05$ (*), $p<0.01$ (**), $p<0.001$ (***) and $p<0.0001$ (****)
