# Supplementary material for: A proportion of CD4+ T cells from patients with chronic Chagas disease undergo a dysfunctional process, which is partially reversed by benznidazole treatment
Source: PLoS Negl Trop Dis. 2021 Feb 4;15(2):e0009059. doi: 10.1371/journal.pntd.0009059 (PMC7888659; doi:10.1371/journal.pntd.0009059)
Supplement: Attachment — Submitted filename: Response to Reviewers PNTD-D-20-01213.doc [file pntd.0009059.s005.doc]

**Response to Reviewers**

**Reviewer #1: Abstract**

1- Asymptomatic and symptomatic are not synonymous of IND and CCC. A patient with the cardiac form may be asymptomatic. Therefore, this point shall be revised, and a unique nomenclature adopted all the manuscript.

As suggested by the reviewer, it has been clarified in the revised version that the patients were classified following Kuschnir nomenclature (referred in detail in the revised text) as Indeterminate (G0) and CCC (G1-G3).

2- The analyzed molecules shall be described in the abstract.

As indicated, the inhibitory receptors (2B4, CD160, CTLA-4, PD-1 and/or TIM-3), cytokines and cytotoxic molecules (IFN-γ, IL-2, TNF-α perforin and/or granzyme B) analyzed in this study are described in the abstract of the corrected manuscript.

The two first sentences of the background have been fused in order to fit the word counting required for the abstract.

3- The conclusion shall be revised, or the data reanalyzed as correlation studies were not performed and there is no analysis of correlation with the severity of the CCC form (mild, moderate, severe).

The conclusion has been revised as indicated by the Referee, indicating in the revised manuscript “A CD4^+^ T cell dysfunctional process was detected in chronic Chagas disease patients, being more exacerbated in those patients with cardiac symptoms.”

The correlation studies reported in this manuscript are carried out between IND (G0) patients and CCC patients with cardiac manifestations (G1-G3). Due to the low number of patients with advanced cardiopathology (5 patients in stage G2 and 1 in stage G3), it is not possible to perform correlation studies between the degree of severity of the disease among the CCC patients and the expression profile of the molecules under study.

4- Was the beneficial effect of Bz transient? Is it?

The results presented in the manuscript support that the modifications in the expression of the molecules under study (mainly detected in the co-expression of the inhibitory receptors) were observed at short-term after treatment (9-12 months) and were maintained up to 24-48 months post treatment which was the end time of follow-up of the patients. Consequently, we can conclude that the beneficial effect of Bz is not transient as it is maintained at least up to 24-48 months post treatment. This fact has been mentioned in the discussion section of the revised manuscript (lines 693-699).

5- A more general conclusion shall be proposed.

As indicated by the Referee (also in Abstract question 3) the conclusion has been rewritten and improved following the Referee recommendations.

**Introduction**

1- Line 77: Conceptually, is Chagas disease spread or infected persons?

For clarification, the refereed sentence has been rewritten as follows: “In addition, as a result of migratory flows, Chagas disease has spread to non-endemic countries”.

2- The description of the data is superficial. The literature findings supporting the selection of the biomarkers used shall be better sustained, the analyzed phenotypes described.

As suggested by the Referee, the analyzed phenotypes have been described in the introduction section and references supporting the selection of these biomarkers included (lines 86-90, 101-104).

3- As the present study is not the first to approach the effects of Bz therapy on T cell functional biomarkers, therefore some data should be presented in this section.

Following Referee 1 indication, data regarding the effects of Bz therapy on T cell functional biomarkers as well as new references, have been included in the introduction section of the revised manuscript (lines 110-115).

**Material and Methods**

1- Patients are neither material nor methods. A section of Description of the cohort is required, including the ethics statements.

In accordance with Referee 1, the "Study Population" including statements of ethics, has been placed in a separate section from that of material and methods under the caption “Study population, material and methods” as also suggested by Referee 3. As indicated by the Referee 1, the cohort and the parameters used to classify the patients included in the study have been described in detail (Lines 141-154).

2.- Were all the studied samples submitted to the same protocol?
Collected, cells separated, frozen? Were samples matched? For example, time kept frozen?

All blood samples were collected and maintained to room temperature. PBMCs were purified between 16 and 18 hours after collection and subsequently frozen in liquid nitrogen following the same protocol. The pretreatment and posttreatment samples from each one of the patients were thawed, processed and analyzed at the same time, verifying that viability was greater than 85% in all cases. These indications have been included in Material and Methods section (Peripheral blood mononuclear cell isolation paragraph, lines 186-191).

3.- How many simultaneous labelling were performed? What were the combination of staining? (ex CD3, CD4, x, y,z, w). What was the apparatus used? how many cells How were the samples analyzed and the software used?
The gating strategies shall be described [for example, singlets (R1), dead-cell exclusion (FSC-A × SSC-Lin, R2), CD3 × CD4 dot plot, so on]

As indicated by the Referee, the method and protocol employed for simultaneous labelling, the combination of antibodies used for cell staining, the apparatus employed to acquire cells, the number of acquired cells, how the samples were analyzed and the softwares employed for analyses have been extensively described in the revised manuscript (“Intracellular staining to evaluate the production of cytokines and cytotoxic molecules” and “Surface staining to evaluate the expression of inhibitory receptors” sections).

As indicated by the Editor and Referee 1, a gating strategy has been included as Supplementary Figure 1 in the revised version of the manuscript. It is referred in the manuscript (lines 263-264 and 268-272) and described in detail in the legend to Supplementary Figure 1.

4- Line 189: Why was this time used? The authors did not justify the choice of the time point (10 hours) used in cells cultures.

The stimulation time was chosen based on the literature and our reported previous work. This fact has been mentioned in the “material and methods” section (lines 240) and the correspondent references included in the revised manuscript.

5- Do all the biomarkers show the same kinetics of expression/regulation/modulation? Is 10 hs the appropriate time to study all the biomarkers?

A single time has to be analyzed due to the limitation on the cell number isolated from the blood collected from the patients (following the protocol approved by the Ethics Committee) and the elevated number of cells which have to be acquired to analyze all the biomarkers under study. The time selected has been widely used in the literature. Some of these manuscripts have been included in the revised version of the manuscript. These facts have been mentioned in the revised version of the manuscript (lines 240-244 of Material and methods section).

On the other hand, the study of the expression of all the biomarkers analyzed has allowed us to detect the co-expression of several co- inhibitory molecules and its association with the polyfunctional response at the same time.

6- Line 192: The use of anti-CD28 is clear. But why CD49d was used? (the choose of these antibodies shall be support by literature)

As previously reported, stimulation using both CD28 and CD49d co-stimulators greatly improves the expression of IFN-γ and IL-2 cytokines by human CD4+ memory T cells in cell cultures *in vitro*. Furthermore, it has been also reported that addition of these two co-stimulators enhances the activation of the T. cruzi antigen-specific T cells. For clarification, in the new version of the manuscript two references supporting the use of these molecules have been included (line 247).

7- Line 198: It is not clear: 10 hs of stimulus and 9 hs with brefeldin? Why this?

As indicated, the correspondent sentence has been rewritten for clarification as follows: “…to detect the production of cytokines and cytotoxic molecules, and after stimulation with *Tc*SA (1 µg/mL) for 1 h at 37 ° C (new reference 30), the cells were cultured for additional 9 h in the presence of brefeldin A (1 µg/mL) and monensin (2 µM)” (lines 245-247).

8- How was followed the effectiveness of Bz effects on parasite load? These data shall be presented. Any effects on clinical signs?

The effectiveness of Bz effects on parasite load was analyzed by PCR in almost all patients pre- and post- treatment. Following the indication of the Referee, these results have been included and discussed in the revised version of the manuscript (lines 578-586 and 752-757, respectively) as well as added the correspondent methodology (lines 193-201). In addition, in the Result section it has been indicated that “Throughout the study, no clinical changes were observed in the patients” (lines 585-586).

**Results**

1- In general, results shall be better described.

Results section has been carefully revised following the indications and suggestions made by the Referee. New panels have added into two Figures (Figure 1 and Figure 3); two Supplementary Figures (Supplementary 1 and Supplementary 3), a Table (Table 1) and a Supplementary Table (Table S1) have been incorporated in the corrected version of the manuscript.

2- The sequence of cytometry analysis and the representative profiles of the FACS analysis shall be shown for all the studied profiles (singlets, R1, dot plots).

As indicated by the Editor and Referee 1, a new Figure has been included (Supplementary Figure 1) in the revised version of the manuscript including the representative profiles of the FACS analyses. It is referred in the manuscript (lines 263-264) and described in detail in the legend to Supplementary Figure 1 (lines 1052-1063).

In addition, representative plots of flow cytometry for the main findings are also included in the corrected manuscript as Figure 1, panel C and Figure 3, panel B and detailed in the correspondent Figure legends (lines 358-360 and 424-427, respectively).

3- Are all data generated of the analyses of results obtained after antigen stimulation (antigen + CD28/CD49d)? Were the results obtained after anti-CD28/CD49d stimulation considered to express the final data (graphs)? Are there differences of results according to these two stimulations?

For clarification, it has been mentioned in the Material and Methods Section (lines 272-276), that “The percentage of expression of each molecule was calculated using FlowJo 9.3.2 software (Tree Star, Ashland, OR) by subtracting the percentage of the background (condition without stimulus plus CD28 and CD49d) from the data obtained after the condition of stimulation with *Tc*SA plus CD28 and CD49d”.

All stimulations with *T. cruzi* antigens (*Tc*SA) were carried out in the presence of CD28 and CD49d as previously described. Two References supporting this method have been included in the revised version (line 247). Indication of how the data are represented in the graphs has been included in Figure 6 legend (lines 567-570), and Supplementary Figures 2 and 3 (lines 1070-1074 and 1084-1086, respectively).

4- The legends of the figures shall be improved, describing the stimulators, time of stimulus, way of calculation and statistical method used.

All Figure legends have been carefully revised and improved describing in all cases the stimulators, time of stimulus, way of calculation and statistical method used.

5- All the description using IND/asymptomatic and CCC/symptomatic shall be revised (abstract, introduction, results, legends, discussion). The authors should consider that absence (IND) or presence of signs of cardiac disease (CCC) after exams; the classification of symptomatic or asymptomatic is based on descriptions of the patient. A patient with the cardiac form of Chagas disease may auto declare to be symptomatic.

As suggested by the Reviewer, it has been clarified throughout the entire text that patients were classified following Kuschnir nomenclature (referred in detail in the revised text) as Indeterminate (G0) and CCC (G1-G3).

6- Figure 6: The pie charts are remarkably interesting and shall be maintained. However, the frequencies of cytokines and the profiles of cytotoxic markers are required and should be presented (as the previous figures), allowing the comparison with other findings (including the literature). Representative plots are also needed to support the findings.

As suggested by the Referee, a new Figure (Figure Supplementary 3) has been included in the revised version of the manuscript. Thus, the new Figure includes the data regarding the functional capacity of *T. cruzi*-specific CD4^+^ T cells (frequencies of cytokines and the profiles of cytotoxic markers) from indeterminate and cardiac chronic Chagas disease patients, before and after treatment. These data have been described in the Result section (lines 518-527) and discussed in the Discussion section of the revised manuscript (lines 679-693).

7- Is there a relation with the profiles and CCC severity (mild/ moderate, severe)- correlation graphs may help to clarify this question.

Due to the number of patients with advanced cardiology (5 patients in stage G2 and 1 in stage G3), it is not possible to perform correlation studies between the degree of severity of the disease and the expression profile of the molecules under study.

**Discussion**

1- Line 486/487: To state this correlation (a positive correlation between the degree of exhaustion and the severity of the pathology was found), the study shall be performed.

2- After the analysis and correlation analyses, the discussion shall be rewritten.

In agreement with the Referee, the correspondent sentence has been rewritten indicating that “…. our results show the highest coexpression level of inhibitory receptors in CD4^+^ T cells was observed in chronic Chagas disease patients with cardiac disorders, whose also presented a predominantly monofunctional antigen-specific T cell response. Thus, the CD4^+^ T cell population from the CCC patients showed a higher exhaustion status that those from indeterminate patients”, (lines 613--617 of the revised version).

The comparative analyses carried out in the present study were performed considering healthy donors, patients with Chagas disease in the IND and CCC phases, before and after treatment. The data supporting these finding are stated along the text (“Impact of benznidazole on the expression and coexpression of inhibitory receptors in the population of CD4^+^ T cells from chronic Chagas disease patients”, lines 382-383).

In addition, as answered to Reviewer 1, question 7, due to the low number of patients with CCC (5 patients in stage G2 and 1 in stage G3), it is not possible to carry out correlation studies between the degree of severity of the disease in patients with advance cardiopathology with the expression profile of the molecules under study. However, with the intention of extending the correlation analyses, it has been carried out a correlation study between the expression of the different inhibitory receptors under study considering all the patients with chronic Chagas disease at the different time points [pretreatment (T0) and 9-12 (T1) and 24-48 (T2) months post benznidazole treatment]. The obtained data are included in a new Table (S1 Table). Description of the results is included in the Result section (lines 405-408).

3- The discussion shall consider the temporal and compartmentalization of the immune response.

Following the indication of the Referee, the temporal and compartmentalization of the immune system have been discussed. Thus, two paragraphs have been added to the discussion section, lines 667-678 and lines 693-704).

5- The putative modulatory effects of Bz shall be better discussed and supported.

Following Referee suggestion the modulatory effect of benznidazol has been better discussed in the discussion section of the revised version of the manuscript (Lines 714-722).

Conclusion and limitations should be added.

As indicated above by the Referee, the conclusion has been revised and rewritten. The potential limitations of the study such as number of CCC patients at the G3 stage are referred along the text.

**General comments:**

The cohort and the parameters used to classify the patients shall be better described.

The effects of Bz on parasite load shall be shown and the correlation with the modulation of inhibitory molecules or reduction of the frequency of cells expression these receptors.

The experimental protocols shall be better described.

The results shall be better shown, including representative profiles of FACS analysis.

The discussion shall consider the temporal and compartmentalization of the immune response.

The putative modulatory effects of Bz shall be better discussed.

All the general comments raised by Referee 1 have been carefully addressed and the different questions answered as detailed above.

**Reviewer #2:**

.- The objectives of the study are clearly articulated with a clear testable hypothesis stated, the study design is appropriate to address the stated objectives, the population is clearly described and appropriate for the hypothesis being tested, the sample size is sufficient to ensure adequate power to address the hypothesis being tested. My only concern about the methods is that T2 time of blood collection involves 24 to 48 months after treatment. This is such a long time variation. The authors should explain why is that.

The aim of the study was to evaluate the effect of the benznidalole on the immune response of CD4^+^ T cells at short time posttreatment and whether the changes produced are temporarily maintained over time. For this reason we considered interesting to evaluate the immune response at short and long time posttreatment. This consideration has been discussed in the revised version of the manuscript (lines 667-678 and 689- 695).

.- Also, can you describe better your antigen? Is it excreted and secreted products of trypos?

As indicated by the Referee, the *T. cruzi* antigen employed for cell stimulation has been described in detail in the revised version of the manuscript (211-220).

.-The analysis presented matched the analysis plan and the results are clearly and completely presented. The figures are of good quality, with exception of Fig 5.

Figure 5 has a word in Spanish (leyend) which should be translated to English. Also, I would love to see this figure bigger. The small size is not helping in appreciating the data. Maybe you could divide it in 5 graphs, according to the number of molecules expressed, or find another way of representing it.

As indicated by the Referee, Figure 5 has been divided in three panels to show the frequency of CD4^+^ T cells co-expressing five and four (panel A), three (panel B), two and one (panel C) inhibitory receptors. The Figure size is now bigger and the Spanish word has been deleted.

.- I have concerns about the author summary, though. In line 54 the authors state that "*Trypanosoma cruzi* infection triggers several immune mechanisms in the host that are mostly inadequate to achieve the total clearance of the parasite". This is clearly the view of the authors. This reviewer thinks you should change it to a more scientific, impersonal description of what happens: "the immune mechanisms in the host that don't result in a total clearance of the parasite", or something like that.

The same is suggested for the word "superior" (line 60). It could be changed for something more accurate or just suppressed.

As indicated by the Reviewer 2, the first sentence of the author summary has been rewritten as suggested: “*Trypanosoma cruzi* infection triggers several immune mechanisms in the host that do not result in a total clearance of the parasite, the persistence of which leads to the chronicity of Chagas disease” (lines 52-54). As also indicated the word “superior” has been replaced by “enhanced” (line 58).

A question arises and could enrich your discussion.

.- Is the decline in the co-expression of inhibitory receptors really advantageous for the host? Isn't a regulated response ideal? What if the cells in the Benznidazole-treated indeterminate patients are now more capable of responding and, in an attempt to kill the remaining parasites, also damage the organs and development of symptomatic chronic disease is now triggered?

Following the Referee suggestion, and to enrich the discussion, a new paragraph discussing the questions raised by the Referee has been included in the discussion section of the revised manuscript (lines 762-770).

.- Are these patients been followed? Will they progress slower than untreated indeterminate patients or not progress to disease at all?

Untreated patients to allow comparative analyses regarding the progression of the sickness are not available due to the Ethics Guideline from the Ethics Committee of the Hospital which makes obligatory to offer and to provide the treatment to all patients with a positive serology for Chagas disease. This fact has been referred in the revised manuscript (Lines 151-154).

.- I miss data showing parasite load on these patients on times T0, T1 and T2. May be a PCR in the blood. I know the blood is not so reliable to show parasite load, but it's worth trying.

As it has been also answered to Referee 1, the effectiveness of Bz effects on parasite load was analyzed by PCR in almost all patients pre- and post- treatment. Following the indication of the Referees 1 and 2, these results have been included and discussed in the revised version of the manuscript (lines 578-586 and 752-761, respectively) as well as the correspondent methodology (lines 193-201).

.- The authors must be familiar that it's been suggested that the host organism's goes for maintaining a chronic disease for homeostatic purposes. Too low response results in death with high parasitism. Too big response results in death by immunopathology. So, the body goes for a middle term. Balance. Of course there is a price to be paid in order to keep that population alive for the next generations, and this price happens to be 25% of the individuals dying at the end of the road with cardiac failure. Inhibitory receptors and other regulatory mechanisms are also a means to achieve a balance between low level immunity and low level parasitism. There is this fear that intervening with these regulatory mechanisms in asymptomatic, presumably balanced, patients, could somehow move the scale towards enhanced inflammation and, therefore, disease.

As indicated by the Referee and answered above, in the present manuscript it has been discussed (lines 762-770) the relevance of the homeostasis of the immune system to mediate an equilibrium that result in the elimination of the parasite avoiding an exacerbated immune response that could produce organ damage.

In addition, it has been also discussed the implications of the therapies aimed at blocking inhibitory pathways (lines 770-776).

.- Which brings me to another suggestion. Please discuss the mechanism whereby Benznidazole treatment causes the decline on the co-expression of the inhibitory receptors. Is it because of reduced parasitism? Can you show the reduced parasitism by PCR?

To elucidate the mechanism whereby Benznidazole treatment causes the decline on the co-expression of the inhibitory receptors will require further studies. We estimate that the persistence of the parasite load led to an exhaustion of the functional specific response of the T cells. Consequently, it is expected that a drop in the parasite load will recovery the multifunctional response partially associated to a drop of the coexpression level of the inhibitory receptors. This matter has been discussed in the correspondent section of the revised manuscript (lines 757-761).

PCR was carried out in almost all patients pre- and post- treatment. Following the indication of the Referees 1 and 2, these results have been included and discussed in the revised version of the manuscript (lines 578-586 and 752-757, respectively) as well as added the correspondent methodology (lines 193-201).

.- On the other hand, if proved that the decline in the expression of inhibitory receptors is actually beneficial, so each and every asymptomatic patient should be treated as soon as the infection is diagnosed.

So, in this manuscript, you beautifully show the association between clinical form and expression of these inhibitory receptors. It remains to be shown the real contribution of these "more capable" cells for a symptom-free form of the infection. Would they be capable of totally clearing the parasite?

I think all of those points should be addressed in your discussion in a concise way. You've done that with regard of perforin and granzyme positive CD8 T cells and you can extend the discussion to the inhibitory receptors.

The data reported here show that treatment induces a fast drop in the parasite load that is maintained for a long time (all patients had a negative PCR result for parasite detection at 24-48 months posttreatment) and a decrease in the expression of the inhibitory receptors. A longer time of follow up of the patients will definitively answer this question. Several authors have also reported negative PCR results after treatment in most patients, although not all of them had a parasitic cure. There is a consensus and recommendation of treating to the IND patients. These aspects are referred throughout the manuscript

.- Another interesting point is the one that arises on line 111-114: "Signs of senescence, the later stages of differentiation, the effects of immunoregulatory mechanisms and the expression of inhibitory receptors associated with more severe forms of Chagas disease have also been described in the CD4^+^ T cell population". How is that in the symptomatic individuals we have worse immunopathology even with all that? Is this the result of increased, uncontrolled parasitism? Can it be shown? It can be shown in the animal model, perhaps. Of course this reviewer don't think you need to show this, but it's an interesting point to be discussed.

The patients with more severe forms of Chagas disease don't necessarily have an increased, uncontrolled parasitism. Probably the not complete competence of the immune system in these patients (probably associated with other factors such as the tropism of the parasite, among others) allows the parasites to invade certain tissues where they produce tissue damage (including damage caused by inflammatory processes) that entails the presence of the symptomatology.

We agree with the referee that this is a very interesting and relevant topic.

**Reviewer #3:**

**Specific comments:**

- In the Abstract “Conclusions/Significance” I suggest excluding the statement that cell dysfunctional process was correlated with the severity of the pathology, since this correlation analysis was not performed in the manuscript.

As suggested, the conclusion of the abstract has been revised as indicated by the Referees indicating “A CD4^+^ T cell dysfunctional process was detected in chronic Chagas disease patients, being more exacerbated in those patients with cardiac symptoms.”

- I suggest throughout the text substitute the term indeterminate for asymptomatic.

For clarification, and based on there are patients with the cardiac form whose may be apparently asymptomatic (as indicated and suggested by Referee 1), in the revised version of the manuscript a unique nomenclature have been adopted to standardize the clinical definition of the chronic Chagas disease. Thus, patients have been referred according to Kuschnir nomenclature (referred in detail in the revised text) as Indeterminate (G0) and CCC (G1-G3).

- I suggest substituting the title of the topic Material and methods by Population, material and methods.

As suggested by the Referee, the referred topic has been substituted as indicated: “Study population, material and methods”.

- In the topic Study subjects, the authors should be inserted the demographic data (age ranging and gender) from patients and whether they present any co-morbidities (eg. hypertension, diabetes, coagulopathies, etc).

As indicated by the Referee, it has been stated in the revised text of the manuscript the demographic data from patients (age ranging and sex), (lines 138-145 and 149-151 and Table 1), and the fact that those patients with any co-morbidities (eg. hypertension, diabetes, coagulopathies, etc) were excluded from the study (lines 145-146).

- Why the authors opt to use the Y strain of *T. cruzi* to obtain the parasite soluble antigens?

The *T. cruzi* Y strain has been employed in the laboratory for several analyses that required antigen stimulation of T cells. Previous studies carried out in the laboratory using infection models experimentally infected with different strains of *T. cruzi* showed a similar immune response when the mice splenocytes were stimulated with soluble proteins isolated from any of the *T. cruzi* strains under study (Reference 21 of the original manuscript and 28 in the revised version).

- The concentration of *Tc*Sa (1ug/mL) employed in the short-term *in vitro* antigen stimulation of PBMC seems very low. Please, clarify this choice.

The circulating parasite load is very low during natural chronic infection and consequently the protein concentration that is seen by the immune system of the host is also low. To *in vitro* mimic what happens in natural infection, a low amount of parasite proteins should be used. The *Tc*SA concentration employed in the present manuscript for CD4^+^ T cell stimulation has been previously employed in several previous works which were cited in the original manuscript. In addition, in the corrected version of the manuscript, new references of publications reported by our group and other research groups who use the referred *Tc*SA concentration, have been included (references 30 and 31, lines 252-254).

- For the flow cytometry assays is not clear what was the cytometer used to acquire the samples, the software used to analyze the data, and the number of events acquired/sample. Please, clarify these issues in the Material and Methods.

As indicated by the Referee, the cytometer used to acquire the samples, the software used to analyze the data, and the number of events acquired have been clarified in the Material and Methods section. For this purpose, a new paragraph including the required information has been included in the revised version of the manuscript (lines 268-279).

- Regarding the gating strategy used to analyze the data, I suggest including a complementary figure to present in details this strategy using representative density plots.

As indicated by the Referees 3, a gating strategy has been included as Supplementary Figure 1 in the revised version of the manuscript. It is referred in the manuscript (lines 263-264) and described in detail in the legend to Supplementary Figure 1.

- The number of patients mentioned in the Results usually is not the same described in the Study subjects topic. Please, clarify.

The number of patients under study in each analysis was indicated in the original manuscript, as referred by the Referee. A small variation in the number of patients in each analysis is due to the limitation on the cell number isolated from the blood collected from the patients (following the protocol approved by the Ethics Committee) and the elevated number of cells which have to be acquired to analyze all the biomarkers under study. For clarification, this fact is stated in the revised version (lines 240-244).

- In general, for the results, there are a large variability among the values in many parameters evaluated. Are the data responsible for this variability provided by the same patients? Please, clarify this issue in the text.

For clarification, as indicated by the Referee, a new sentence has been included in the revised manuscript (lines 311-314) indicating that evaluation of the variability of the data was not associated to a particular patient as deduced from the analysis of the outliers of the variables under study.

- Considering the data presented in the Figure 3, did the authors performed any correlations between expression of inhibitory receptors and distinct time points after Bz treatment? This analysis may reinforce the findings obtained using a categorical analysis.

As suggested, to determine significant correlations between the evaluated parameters, a correlation study has been carried out between the expression of the different inhibitory receptors under study and the different time points (T0, T1 and T2) after treatment. The results obtained after a Spearman correlation analysis showed a positive correlation between the modification of the expression level of the five inhibitory receptors evaluated and the time-course after treatment. Obtained data have been included in a new Table (S1 Table), referred in the Result section (lines 405-408) and discussed in the discussion section (lines 695-699).

- The statement about the Figure 5 presented in the text, lines 367 to 370 is confuse. Please, clarify the issue considering the magnitude of increase/decrease in the frequency of inhibitory receptors in asymptomatic patients.

As indicated by the Referee 2, Figure 5 has been divided in three panels to show the frequency of CD4^+^ T cells co-expressing five and four (panel A), three (panel B), two and one (panel C) inhibitory receptors.

For clarification, the Figure 5 legend has been rewritten indicating “The subsets of cells that after benznidazole treatment reduced or increased the percentages of expression with statistically significance are indicated with red and green asterisks, respectively.”

- In the Figure 6, before Bz treatment, the data demonstrated that the proportion of cells expressing 1 or coexpressing 2, 3 is predominant and there are distinct profiles in these proportions in the comparison between asymptomatic and CCC groups. After treatment, these proportions acquired profiles more homogeneous between both groups. These findings must be more broadly addressed in the Discussion.

As indicated by the Referee, in the discussion section of the corrected manuscript, a paragraph discussing these issues has been added (lines 679-693).

Moreover, the percentage of CD4^+^ T cells expressing each one of the molecules under study has been shown in a new Figure (S3 Fig). The results show substantial differences in the expression pattern of particular molecules in cells from IND versus CCC. These results are referred in Result section (lines 518-527) and discussed in discussion section (lines 684-693) of the revised manuscript.

- In some circumstances, the text of Discussion is very speculative, and the authors did not show sufficient data to fundament the statements: e.g. Lines 479-480 - These findings could indicate that a fraction of the CD4^+^ T cell population undergoes a T cell exhaustion process; Lines 486-487 - Thus, a positive correlation between the degree of exhaustion and the severity of the pathology was found. Please, revise them.

Following the Referee indications, the sentence of Lines 479-480 of the original paper has been deleted. In addition the referred sentence of Lines 486-487 has been rewritten as follows: "Thus, the CD4^+^ T cell population from the CCC patients showed a higher exhaustion status that those from indeterminate patients” (Lines 616-617 of the revised manuscript).

- The limitations of the study must be stated in the Discussion.

The potential limitations of the study such as number of CCC patients at the G3 stage, not having the same number of patients in all assays due to the number of cells isolated from the patients, etc., are referred along the text.

.- In the lines 406-407, the acronym for interferon-gamma (IFN-y) is incorrectly presented in the text as INF-. Please, revise it.

The acronym for interferon-gamma has been corrected.
